# Genomic prediction of disease occurrence using producer-recorded health data: a comparison of methods

**DOI:** 10.1186/s12711-015-0093-9

**Published:** 2015-05-08

**Authors:** Kristen L Parker Gaddis, Francesco Tiezzi, John B Cole, John S Clay, Christian Maltecca

**Affiliations:** North Carolina State University, Raleigh, 27695 NC USA; Animal Genomics and Improvement Laboratory, Agricultural Research Service, USDA, Beltsville, 20705-2350 MD USA; Dairy Records Management Systems, Raleigh, 27603 NC USA

## Abstract

**Background:**

Genetic selection has been successful in achieving increased production in dairy cattle; however, corresponding declines in fitness traits have been documented. Selection for fitness traits is more difficult, since they have low heritabilities and are influenced by various non-genetic factors. The objective of this paper was to investigate the predictive ability of two-stage and single-step genomic selection methods applied to health data collected from on-farm computer systems in the U.S.

**Methods:**

Implementation of single-trait and two-trait sire models was investigated using BayesA and single-step methods for mastitis and somatic cell score. Variance components were estimated. The complete dataset was divided into training and validation sets to perform model comparison. Estimated sire breeding values were used to estimate the number of daughters expected to develop mastitis. Predictive ability of each model was assessed by the sum of *χ*^2^ values that compared predicted and observed numbers of daughters with mastitis and the proportion of wrong predictions.

**Results:**

According to the model applied, estimated heritabilities of liability to mastitis ranged from 0.05 (*SD*=0.02) to 0.11 (*SD*=0.03) and estimated heritabilities of somatic cell score ranged from 0.08 (*SD*=0.01) to 0.18 (*SD*=0.03). Posterior mean of genetic correlation between mastitis and somatic cell score was equal to 0.63 (*SD*=0.17). The single-step method had the best predictive ability. Conversely, the smallest number of wrong predictions was obtained with the univariate BayesA model. The best model fit was found for single-step and pedigree-based models. Bivariate single-step analysis had a better predictive ability than bivariate BayesA; however, the latter led to the smallest number of wrong predictions.

**Conclusions:**

Genomic data improved our ability to predict animal breeding values. Performance of genomic selection methods depends on a multitude of factors. Heritability of traits and reliability of genotyped individuals has a large impact on the performance of genomic evaluation methods. Given the current characteristics of producer-recorded health data, single-step methods have several advantages compared to two-step methods.

## Background

Genetic selection has been very successful in achieving increased production in dairy cattle. Consequently, corresponding declines in fitness and fertility have been documented [1]. Fitness and fertility traits are more difficult to select for, since they have low heritabilities and are influenced by various non-genetic factors. Improvement of functional traits through genomic selection is an appealing tool because the changes can be considered long-lasting. Currently, genomic selection methodologies are widely investigated and implemented in dairy cattle breeding [2,3], as well as for other species [4,5]; however, much of this research was aimed at traditional traits, such as those related to production [2,6].

In 2001, Meuwissen et al. [7] showed that all available molecular markers could be used to predict genomic values for quantitative traits. This article introduced two Bayesian procedures to estimate genomic values, termed BayesA and BayesB, which have now been expanded upon and are collectively referred to as the “Bayesian Alphabet” [8]. These multi-stage methods estimate individual marker effects using both phenotyping and genotyping data. In a typical multi-stage genomic procedure, such as that described by VanRaden [9], traditional breeding values are calculated using best linear unbiased prediction (as opposed to best linear unbiased n) (BLUP) methodology [10] for animals with genotyping information. Estimated breeding values can then be deregressed to remove bias and to account for heterogeneous variances, and are used as “pseudo-phenotypes” (dEBV) [11]. Performance of response variables has been shown to depend on heritability of the trait, number of daughters per sire, number of animals genotyped, and type of statistical model applied in the simulation studies [12]. This is particularly problematic when working with categorical traits on a liability scale, in addition to having low reliabilities. Genomic effects for each marker can be estimated and used to calculate direct genomic values (DGV) for each genotyped animal. The DGV can be further combined with traditional measurements of merit, including parent average (PA) and estimated breeding value (EBV), to calculate a breeding value that accounts for phenotype, pedigree, and genotype information [9].

A single-step method was proposed as an alternative to multi-stage approaches [13-15]. It is now more commonly referred to as single-step genomic BLUP, but for comparative purposes to two-stage methods, we will refer to it only as single-step herein. The single-step procedure replaces pedigree (**A**) and genomic (**G**) relationship matrices with a blended **H** matrix [15,16] that combines information from both **A** and **G**. This permits the simultaneous estimation of breeding values while accounting for population structure, and can also account for systematic effects such as genomic pre-selection bias [17]. Substitution of **A** by matrix **H** enables this method to be easily expanded to more complex models, such as multivariate or random regression models [18].

One goal when incorporating genomic data is to increase the reliability of estimated breeding values. Typically, the reliabilities of genetic evaluations of health traits are low; thus, these traits may benefit greatly from including genomic data. This has been previously demonstrated with producer-recorded health data on six common health issues [19]. High-density genomic data may improve reliability even more by improving predictions. Improvement in reliability is a key component to the success of genomic selection, but improvement cannot be evaluated for the same population as that used to develop the prediction model [20]. To evaluate the performance of genomic evaluation methods, cross-validation is often performed. A training population is used to estimate marker effects from animals with both genotypes and phenotypes. Estimated marker effects are then used in the validation population to evaluate the prediction model using trait phenotypes. Data are split into training and validation groups using one of several methods, such as splitting based on birth year or relationship.

Comparison between the performance of two-stage and single-step methodologies is difficult regardless of the trait. Two-stage methods provide an estimate of DGV, which should ideally be blended with other sources of information (i.e., pedigree data, parent average) before calculating a measure of reliability. Numerous approaches to estimate reliability of two-stage estimates have been used (e.g., [12,21,22]). The single-step method combines genomic data and pedigree data within the analysis. An approximation method to estimate reliability of single-step results has been developed [23]. Predictive ability of future records can be assessed as opposed to directly comparing the different methodologies. Cross-validation of a model’s predictive ability has already been applied on dairy cattle data, including for functional traits such as number of inseminations to conception [24], daughter longevity [25], and mastitis [26].

Methods to extend two-stage methods from univariate to multivariate models are currently being investigated. Calus and Veerkamp [27] used simulated data to investigate the performance of three marker-based models in multiple-trait analyses. They found that accuracies increased, in particular for young animals with no phenotype, when using a multiple-trait model compared to a single-trait model. To expand upon these results, Jia and Jannink [28] investigated three multivariate linear models using both simulated and real data. Their results indicated that prediction accuracy for lowly heritable traits could be significantly increased by multivariate genomic selection when a correlated trait with a higher heritability was included. However, currently there is little literature on the implementation of multivariate two-stage genomic models with non-simulated data.

Several studies have analyzed functional and production traits using genomic data [22,29], although many of these were conducted outside the U.S. The fact that there is only a limited amount of research on the genomic evaluation of health traits in the U.S. may be due in part to a lack of documented phenotypes. Producer-recorded health information from U.S. dairies may be able to fill this gap and provide health-related phenotypes. The objective of this study was to investigate the predictive ability of two-stage and single-step genomic methods applied to health data collected from on-farm computer systems in the U.S. Implementation of univariate and bivariate models was investigated using BayesA and single-step methodologies for mastitis and somatic cell score (SCS). A BayesA model was chosen since this is the method being implemented in the U.S. Mastitis was selected from the producer-recorded health data because of the large impact it has on the dairy industry. Somatic cell score provides a corresponding trait that has a higher heritability and is commonly used as an indicator trait for mastitis. Currently, in the U.S., SCS and an udder composite are incorporated as indicators of mastitis in genetic evaluations. Traits included in the udder composite include more structural features such as udder depth, teat placement, and rear udder height [30]. Greater and more rapid improvement may be possible if direct records of mastitis were used as opposed to these indirect measures.

## Methods

### Data

Producer-recorded health data from U.S. dairy farms were available from 1998 through 2012. Mastitis events were assigned to a lactation, with lactations beginning with a calving. Only the first mastitis occurrence per lactation was included for each cow. Occurrences of mastitis from first parity cows were selected for analysis. Minimum and maximum reporting contraints were imposed on the data by herd-year. Lactations lasting up to 400 days postpartum were included in the analyses. Additional general editing was applied to the data as described by Parker Gaddis et al. [31]. To ensure that sires included in the analyses could be equally compared across analyses, additional restrictions were placed on the data. Sires were required to have at least 15 daughters with mastitis records. The number of daughter records per sire ranged from 17 to 1409, with a median number of daughters per sire equal to 87. Older sires may have had granddaughters with phenotype records. If this occurred, these records were removed to ensure that all sires were represented equally. All analyses were also performed with datasets without applying the additional daughter restrictions. This was done such that performance in a more typical health dataset (more sires with fewer daughters) could be evaluated. The data without applying daughter restrictions will be referred to as *DATA*_*full*_; data with daughter restrictions will be referred to as *DATA*_*dtr*_ throughout.

Descriptive statistics for the data are in Table 1. Before applying daughter restrictions, *DATA*_*full*_ included 97 310 mastitis records from first parity cows. These cows were from 10 549 sires and 11 040 maternal grandsires. *DATA*_*dtr*_ included 26 510 mastitis records from first parity cows. Records were from 177 sires and 4328 maternal grandsires. Records included 52 year-seasons and 2210 herd-years. Training and validation datasets were created by splitting each full dataset based on year. Records before 2009 were included in the training data; records for 2009 and later were included in the validation data. This was done to reflect the true accumulation of data that occurs in the dairy industry. Mean lactation incidence rate of mastitis in the full *DATA*_*dtr*_ was estimated at 10.5%. Mean lactation incidence rate of mastitis in training and validation datasets were similarly equal to 10.2% and 13.0%, respectively. Despite the small dataset, these incidence values were similar to those in *DATA*_*full*_, as well as to incidences previously reported in the literature [32-34].

**Table 1:** **Descriptive statistics for full, training, and validation datasets with and without daughter restrictions enforced**

**Data without daughter restrictions**			
	**Full data**	**Training data**	**Validation data**
Years included	1999 - 2012	1999 - 2008	2009 - 2012
Number of cows	97 310	79 147	18 163
Number of mastitis incidences	10 442	8391	2051
Number of sires	10 549	8410	3269
Number of maternal grandsires	11 040	8938	3636
Average number of daughters per sire	9	9	6
Average mastitis incidence	0.107	0.106	0.113
Average mastitis incidence per sire	0.104	0.104	0.113
**Data with daughter restrictions**			
	**Full data**	**Training data**	**Validation data**
Years included	1999 - 2012	1999 - 2008	2009 - 2012
Number of cows	26 510	23 753	2757
Number of mastitis incidences	2771	2422	349
Number of sires	177	153	59
Number of maternal grandsires	4328	3823	909
Median number of daughters per sire	87	91	37
Average mastitis incidence	0.105	0.102	0.130
Average mastitis incidence per sire	0.106	0.100	0.140

Genomic data from the Illumina BovineSNP50 BeadChip (Illumina Inc., San Diego, CA) were available for 7883 sires. Standard filters were previously applied to the marker data, including removing SNPs with minor allele frequencies less than 0.05 and SNPs that were in complete linkage disequilibrium with other SNPs, resulting in a final marker set of 37 506. There were 177 genotyped sires that had at least 15 daughter records in the final dataset. High-density (HD) genotypes were also available for 1371 sires. Similar editing procedures were applied to these data, including removal of SNPs with minor allele frequencies less than 0.05 and SNPs that were in complete linkage disequilibrium. This resulted in a dataset of 281 868 markers for 177 sires with at least 15 daughter records. A full summary of these data is in Table 1.

### BayesA analyses

Traditional EBV were calculated using THRGIBBS1F90 (version 2.104) [35] by fitting the single-trait threshold model below:

$$ \boldsymbol{\lambda} = \boldsymbol{X\beta} + \mathbf{Z_{h}}\mathbf{h} + \mathbf{Z_{s}}\mathbf{s} + \mathbf{e} $$

where ***λ*** represents a vector of unobserved liabilities to mastitis or SCS, ***β*** is a vector of fixed effects including year-season, **X** is the corresponding incidence matrix for fixed effects, **h** represents the random herd-year effect, where $\mathbf {h} \sim N\left (0, \mathbf {I}{\sigma ^{2}_{h}}\right)$ with **I** representing an identity matrix, **s** represents the random sire effect, where $\mathbf {s} \sim N\left (0, \mathbf {A}{\sigma ^{2}_{s}}\right)$ with **A** representing the additive relationship matrix, *Z*_*h*_ and *Z*_*s*_ represent corresponding incidence matrices for the appropriate random effect, and **e** represents the random residual, assumed to be distributed as **e**∼*N*(0,**I**). Residual variance was fixed at 1 for identifiability. A probit link was used to transform event incidence to liability. A total of 100 000 iterations were performed, with the first 10 000 iterations discarded as burn-in for both full and training datasets. Every 10^*th*^ sample was saved to reduce autocorrelation. This resulted in a total of 9000 samples used for post-Gibbs analyses completed using POSTGIBBSF90 (version 3.04) [36], including visual inspection of trace plots and posterior distributions. Convergence was also assessed by calculating Geweke’s convergence statistic [37] with the coda package [38] in R (version 2.15.1) [39]. Variance components, standard deviations, and 95% highest posterior densities were calculated from the resulting posterior distributions. Highest posterior densities were calculated with the coda package [38] in R (version 3.0.2) [40]. Estimated breeding values were calculated by doubling estimated sire effects. Reliabilities of sire EBV were estimated using ACCF90 (version 1.67) [36].

Single-trait BayesA analyses were performed using the GenSel software (version 4.25R) [41]. EBV of mastitis and SCS were deregressed by a function of reliability given by 1/(1−*reliability*), which was scaled to have a mean equal to 1 [29]. A single-trait analysis of mastitis using unweighted EBV was also performed for comparative purposes. All markers were included as predictors in the model, with deregressed EBV as the response variable to predict marker effects. Weights were effectively incorporated as elements of an inverse diagonal matrix of residual variance. The model for univariate analyses of mastitis and SCS is given below:

$$y_{i} = \mu + \sum_{j=1}^{k}z_{ij}u_{j} + e_{i} $$

where *y*_*i*_ is the deregressed EBV for sire *i*, *μ* is the overall mean, *z*_*ij*_ is the genotype of sire *i* at marker *j*, *u*_*j*_ is the effect of marker *j*, and *e*_*i*_ represents random error distributed following $N\left (0, I{\sigma _{e}^{2}}\right)$. A chain of 300 000 iterations with the first 50 000 iterations discarded as burn-in, saving every 100 samples was performed for both the full and training datasets. This resulted in a total of 2500 samples. Accuracy of BayesA analyses were calculated following Saatchi et al. [20], as shown below:

$$\hat{\rho}_{g,\hat{g}} = \frac{\hat{\sigma}_{dEBV, DGV}}{\sqrt{{\sigma^{2}_{g}}\hat{\sigma}^{2}_{DGV}}} $$

where $\hat {\rho }_{g,\hat {g}}$ is the accuracy of DGV, $\hat {\sigma }_{dEBV,DGV}$ is the covariance between dEBV and DGV from the analysis, ${\sigma ^{2}_{g}}$ is the additive genetic variance, and $\hat {\sigma }^{2}_{\textit {DGV}}$ is the variance of DGV. Additive genetic variance was obtained from prior pedigree-based analyses. This calculation of accuracy standardizes the covariance between dEBV and DGV in order to account for heterogeneous variances among sires [20]. Reliability was obtained by squaring this estimate of accuracy.

A corresponding bivariate BayesA analysis was performed on mastitis and SCS data. Pedigree-based EBV were obtained as described above, except that a bivariate model was used in this case. We used the partially modified C code developed by Jia and Jannink [28] to investigate the performance of two-trait BayesA analyses. The model implemented was similar to that of single-trait BayesA analyses described previously. Marker effects in bivariate BayesA analyses were sampled from a multivariate normal distribution following $MVN\left (0, \sum _{a}\right)$ and the variance, $\sum _{a}$, was sampled from an inverted Wishart distribution following *inv*−*Wis*(*ν*,*S*_*n*×*n*_), where *n* equals the number of traits. Number of degrees of freedom (*ν*) was fixed at 4.012 and scale (*S*_*n*×*n*_) was fixed at 0.002 on the diagonal and 0 otherwise.

### Single-step analyses

To perform univariate single-step analyses, preGSf90 (version 1.142) was used to create the inverse blended **H** matrix [42]. A bivariate single-step analysis was also performed using HD genotype data. The blended **H** matrix was incorporated into a threshold sire model using THRGIBBS1F90 (version 2.104) [35]. The model fitted was:

$$ \boldsymbol{\lambda} = \boldsymbol{X\beta} + \mathbf{Z_{h}}\mathbf{h} + \mathbf{Z_{s}}\mathbf{s} + \mathbf{e} $$

where ***λ*** represents a vector of unobserved liabilities to mastitis or SCS, ***β*** is a vector of fixed effects including year-season, **X** is the corresponding incidence matrix of fixed effects, **h** represents the random herd-year effect, where $\mathbf {h} \sim N\left (0, \mathbf {I}{\sigma ^{2}_{h}}\right)$, with **I** representing an identity matrix, **s** represents the random sire effect where $\mathbf {s} \sim N\left (0, \mathbf {H}{\sigma ^{2}_{s}}\right)$ with **H** representing the blended relationship matrix of pedigree and genomic information, *Z*_*h*_ and *Z*_*s*_ represent the corresponding incidence matrices for random effects, and **e** represents the random residual, assumed to be distributed as *N*(0,**I**). The residual variance was fixed at 1 for identifiability. The $\mathbf {A_{22}^{-1}}$ matrix was given a weight of 0.4 using the “TauOmega” option of preGSf90 (version 1.142) [42] to aid in convergence. A chain of 300 000 iterations was completed with 30 000 samples discarded as burn-in. Every 30 samples were saved to reduce autocorrelation. Post-Gibbs analysis and convergence assessment were completed on the 9000 samples with POSTGIBBSF90 (version 3.04) [36]. Posterior means, standard deviations, and 95% highest posterior densities were calculated to obtain estimates of variance components.

A bivariate analysis was also performed using single-step methodology for mastitis and SCS. The model remained comparable to that described above, except that it was expanded to two dependent variables:

$$ \mathbf{Y} = \boldsymbol{X\beta} + \mathbf{Z_{h}}\mathbf{h} + \mathbf{Z_{s}}\mathbf{s} + \mathbf{e} $$

where **Y** represents a vector of liabilities to mastitis as well as phenotypic values of SCS. All other variables were the same as defined previously. The model was fitted using THRGIBBS1F90 (version 2.104) [35]. A chain of 500 000 iterations was completed with 50 000 samples discarded as burn-in. Every 50 samples were saved to reduce autocorrelation in the full dataset; every 100 samples were saved to reduce autocorrelation in the training dataset. This resulted in a total of 9000 samples for the full dataset and 4500 samples for the training dataset. Post-Gibbs analysis and convergence assessment were completed with POSTGIBBSF90 (version 3.04) [36]. Posterior means, standard deviations, and 95% highest posterior densities were calculated as estimates of variance components.

Reliabilities of solutions from single-step analyses were estimated following Misztal et al. [23]. Reliabilities estimated from previously described pedigree-based analyses using ACCF90 (version 1.67) [36] were used as reliabilities calculated without genomic information. Pedigree-based reliability estimates were converted to effective number of records for genotyped animals (*d*_*i*_) as:

$$d_{i} = \alpha\left[1/\left(1 - \mathit{rel}_{p_{i}}\right)-1\right] $$

where *α* is the ratio of residual variance to genetic variance calculated from the pedigree-based analysis and ${rel}_{p_{i}}$ represents reliability of the EBV for individual *i* from pedigree-based analysis [23]. The values of *d*_*i*_ were used to create the diagonal matrix **D**. The inverse matrix **Q** was calculated as:

$$\mathbf{Q_{i}} = \left[\mathbf{D} + \left(\mathbf{I} + \mathbf{G^{-1}} - \mathbf{A^{-1}_{22}}\right)\alpha\right]^{-1} $$

where *G*^−1^ is the inverse of the genomic relationship matrix and $\mathbf {A^{-1}_{22}}$ is the inverse of the pedigree-based relationship matrix between genotyped animals only [23]. Genomic reliabilities for each sire were then estimated as:

$$\mathit{rel}_{g_{i}} = 1 - \alpha q^{ii} $$

where ${rel}_{g_{i}}$ represents approximate genomic reliability and *q*^*ii*^ is the diagonal element of *Q*^−1^ corresponding to the *i*^*th*^ sire [23].

### Model comparison

To perform model comparison, the complete dataset was divided into two subsets based on year of mastitis occurence to represent accumulation of data in the dairy industry. The training dataset included records from 1999 through 2008. The validation dataset included records from 2009 through 2012 and was used to test estimates obtained with the training dataset. This resulted in an approximate 80%-20% split of the data. Editing was applied to the datasets to ensure sires had sufficient daughter records in order to perform fair comparisons. For sires to be included in the validation dataset, they had to have at least 30 daughters with records and for sires to be included in the prediction dataset, they had to have at least 15 daughters with records.

Predictions were performed to compare models. The probability for daughters to develop mastitis for each sire was used to evaluate model predictive ability. Average incidence of daughters developing mastitis was calculated for the validation dataset. These were regressed using a logistic link on EBV calculated from training data using the logistic procedure of SAS (SAS Institute Inc., Cary, NC). Coefficients obtained from the logistic regression model allowed EBV from the training data to be transformed into a probability for daughters to develop mastitis for each sire. The obtained probability was multiplied by the number of observations (daughters) for each sire in the validation dataset to calculate expected number of daughters with mastitis. Predictive ability of each model was assessed using a sum of *χ*^2^ modified from González-Recio et al. [24]. The *χ*^2^ value was calculated for each sire between expected “success” (daughters without mastitis) and “failures” (daughters with mastitis) from EBV based on training data and observed number of daughters with and without mastitis in the validation data, as shown below:

$$\begin{array}{@{}rcl@{}} &\chi^{2}= \left[\left(\text{expected success} - \text{observed success}\right)\right]^{2} \\&\qquad \quad+ \left.\left(\text{expected failures} - \text{observed failures}\right)^{2}\right]. \end{array} $$

Expected values are thus based on calculated probabilities. Observed values were those from the validation dataset. Calculated *χ*^2^ values were summed across sires, resulting in a single *χ*^2^ sum for each model. For model comparison, smaller *χ*^2^ values are preferred. Among all sires, there were 35 sires with records in both training and validation datasets. We recognize that this is a very limited number of sires; however, the strict editing was put in place to ensure that equivalent comparisons could be performed. As previously mentioned, analyses were also performed without enforcing the strict criteria on number of daughters. This allowed performance to be compared between the very confined dataset and a more typical dataset.

Predictive ability of the model was also assessed by calculating the proportion of wrong predictions (**WP**), which was the difference between expected and observed healthy daughters divided by the total number of daughters for each sire. Smaller values are preferred for this metric as well.

Model fit was evaluated using local weighted regression [43], with EBV estimated from the full dataset and average incidence per sire in the full dataset. Regression parameters were calculated with PROC LOESS in SAS (SAS Inst. Inc., Cary, NC). The optimum smoothing parameter was selected based on a corrected Akaike’s information criterion (AICC) [44]. This smoothing parameter determines the number of datapoints within each local neighborhood that affects the complexity of model fit.

## Results and discussion

Posterior means of variance components for full and training datasets for both *DATA*_*full*_ and *DATA*_*dtr*_ are in Table 2 from univariate pedigree-based analyses of mastitis and SCS. Comparisons between *DATA*_*full*_ and *DATA*_*dtr*_ provided very similar estimates of variance components. Heritability of liability to mastitis was greater in *DATA*_*full*_ in most cases. Heritability estimates calculated as the mean of posterior distributions were 0.05 (*SD*=0.02) in both full and training datasets for liability to mastitis in *DATA*_*dtr*_. Highest posterior density 95% intervals for heritability of liability to mastitis using *DATA*_*dtr*_ were (0.02,0.08) for both full and training datasets. Heritability estimates of SCS from *DATA*_*dtr*_, calculated as the mean of resulting posterior distributions, were 0.08 (*SD*=0.01) for both full and training datasets. Posterior means of variance components for full and training datasets from single-step analyses are also in Table 2. Heritability estimates from single-step analyses for liability to mastitis were equal to 0.11 (*SD*=0.03) and 0.06 (*SD*=0.02) for the full and training datasets of *DATA*_*dtr*_, respectively. Heritability estimates from univariate single-step analyses of SCS were equal to 0.18 (*SD*=0.03) for both full and training datasets of *DATA*_*dtr*_, respectively. This was higher than the heritability of SCS estimated with *DATA*_*full*_. Highest posterior density 95% intervals for heritability of liability to mastitis were (0.05,0.18) and (0.02,0.10) for full and training *DATA*_*dtr*_, respectively. Highest posterior density 95% intervals for heritability of SCS were (0.13, 0.24) for both full and training *DATA*_*dtr*_. Posterior means of residual and genetic variance were used to calculate the proportion of variance accounted for by markers in univariate BayesA analyses of mastitis and were equal to 0.156 and 0.121 for the full and training datasets of *DATA*_*dtr*_, respectively. In general, variance component estimates from each dataset and analysis method were very similar. Heritability estimates calculated with *DATA*_*dtr*_ were lower than those calculated previously with a larger dataset [19]; however, they were still within the range of reported values [45,46]. Heritability estimates of SCS were also similar to other reports found in the literature [47-49].

**Table 2:** **Single-trait model variance component estimates (standard deviation)**

	**Mastitis**							
	**Data without daughter restrictions**				**Data with daughter restrictions**			
	**Pedigree-based analysis**		**Single-step analysis**		**Pedigree-based analysis**		**Single-step analysis**	
	**Full data**	**Training data**	**Full data**	**Training data**	**Full data**	**Training data**	**Full data**	**Training data**
${\sigma _{s}^{2}}$	0.02 (0.004)	0.03 (0.004)	0.04 (0.006)	0.05 (0.007)	0.02 (0.006)	0.02 (0.006)	0.04 (0.01)	0.02 (0.008)
${\sigma _{h}^{2}}$	0.49 (0.03)	0.46 (0.03)	0.49 (0.02)	0.46 (0.03)	0.43 (0.03)	0.41 (0.04)	0.43 (0.03)	0.41 (0.04)
${\sigma _{e}^{2}}$	1.0 (0.006)	1.0 (0.007)	1.0 (0.006)	1.0 (0.007)	1.0 (0.01)	1.0 (0.01)	1.0 (0.01)	1.0 (0.01)
*h* ^2^	0.10 (0.01)	0.12 (0.01)	0.10 (0.02)	0.12 (0.02)	0.05 (0.02)	0.05 (0.02)	0.11 (0.03)	0.06 (0.02)
	**Somatic Cell Score**							
	**Pedigree-based analysis**		**Single-step analysis**		**Pedigree-based analysis**		**Single-step analysis**	
	**Full data**	**Training data**	**Full data**	**Training data**	**Full data**	**Training data**	**Full data**	**Training data**
${\sigma _{s}^{2}}$	0.04 (0.004)	0.04 (0.004)	0.07 (0.006)	0.07 (0.006)	0.05 (0.008)	0.05 (0.008)	0.10 (0.02)	0.10 (0.02)
${\sigma _{h}^{2}}$	0.53 (0.02)	0.53 (0.02)	0.53 (0.02)	0.52 (0.02)	0.52 (0.02)	0.50 (0.03)	0.52 (0.02)	0.50 (0.03)
${\sigma _{e}^{2}}$	1.64 (0.008)	1.64 (0.008)	1.63 (0.008)	1.60 (0.008)	1.62 (0.01)	1.62 (0.02)	1.62 (0.01)	1.62 (0.02)
*h* ^2^	0.08 (0.01)	0.08 (0.01)	0.13 (0.01)	0.13 (0.01)	0.08 (0.01)	0.08 (0.01)	0.18 (0.03)	0.18 (0.03)

Estimated variance components include sire variance (*σ* *s*2), herdyear variance (*σ* *h*2), residual variance (*σ* *e*2) and heritability (*h* ^2^) for full and training datasets from pedigree-based and single-step analyses of mastitis and somatic cell score.

Table 3 includes posterior means of variance components for bivariate analyses of full and training data from both *DATA*_*full*_ and *DATA*_*dtr*_. Similar to univariate analyses, variance components between each dataset were very similar. Pedigree-based and single-step analyses are included. Heritability estimates calculated for mastitis in pedigree-based analyses were similar to those with univariate models. The highest posterior density 95% intervals from pedigree-based analysis of liability to mastitis were (0.01, 0.06) for both full and training *DATA*_*dtr*_. Posterior mean heritability of liability to mastitis was higher in the single-step than the pedigree-based analyses, equal to 0.08 (*SD*=0.03) for both full and training *DATA*_*dtr*_. Highest posterior density 95% intervals for heritability of liability to mastitis were (0.03, 0.14) and (0.03, 0.13) for full and training *DATA*_*dtr*_, respectively. Posterior mean heritability for SCS was 0.09 (*SD*=0.02) and 0.10 (*SD*=0.02) in pedigree-based analyses using full or training *DATA*_*dtr*_, respectively. Posterior mean heritability of SCS was also higher in single-step analyses, as shown in Table 4. The higher heritability estimates obtained in the single-step analyses may be a result of bias introduced in tuning the **H** matrix by weighting **G** and *A*_22_ to aid in convergence. Proportions of total variance accounted for by markers in bivariate BayesA analyses of mastitis with SCS were equal to 0.134 and 0.118 for the full and training *DATA*_*dtr*_, respectively.

**Table 3:** **Bivariate model genetic variance component estimates (standard deviation)**

	**Mastitis**							
	**Data without daughter restrictions**				**Data with daughter restrictions**			
	**Pedigree-based analysis**		**Single-step analysis**		**Pedigree-based analysis**		**Single-step analysis**	
	**Full data**	**Training data**	**Full data**	**Training data**	**Full data**	**Training data**	**Full data**	**Training data**
${\sigma _{s}^{2}}$	0.02 (0.003)	0.02 (0.004)	0.03 (0.01)	0.04 (0.01)	0.01 (0.005)	0.01 (0.005)	0.03 (0.01)	0.03 (0.01)
${\sigma _{h}^{2}}$	0.49 (0.02)	0.46 (0.03)	0.43 (0.03)	0.46 (0.03)	0.43 (0.03)	0.41 (0.04)	0.43 (0.03)	0.41 (0.04)
${\sigma _{e}^{2}}$	1.0 (0.01)	1.0 (0.01)	1.0 (0.01)	1.0 (0.01)	1.0 (0.01)	1.0 (0.01)	1.0 (0.01)	1.0 (0.01)
*h* ^2^	0.05 (0.01)	0.06 (0.01)	0.09 (0.02)	0.10 (0.02)	0.04 (0.01)	0.05 (0.01)	0.08 (0.03)	0.08 (0.03)
	**Somatic Cell Score**							
	**Pedigree-based analysis**		**Single-step analysis**		**Pedigree-based analysis**		**Single-step analysis**	
	**Full data**	**Training data**	**Full data**	**Training data**	**Full data**	**Training data**	**Full data**	**Training data**
${\sigma _{s}^{2}}$	0.04 (0.004)	0.05 (0.004)	0.11(0.02)	0.07 (0.01)	0.05 (0.01)	0.05 (0.009)	0.11(0.02)	0.11 (0.02)
${\sigma _{h}^{2}}$	0.34 (0.01)	0.33 (0.01)	0.34 (0.02)	0.33 (0.01)	0.34 (0.02)	0.33 (0.02)	0.34 (0.02)	0.33 (0.02)
${\sigma _{e}^{2}}$	1.67 (0.01)	1.63 (0.009)	1.66 (0.02)	1.63 (0.01)	1.66 (0.02)	1.65 (0.02)	1.66 (0.02)	1.65 (0.02)
*h* ^2^	0.09 (0.01)	0.09 (0.009)	0.14 (0.01)	0.14 (0.01)	0.09 (0.02)	0.10 (0.02)	0.20 (0.03)	0.20 (0.03)

Estimated variance components include sire variance (*σ* *s*2), herdyear variance (*σ* *h*2), residual variance (*σ* *e*2) and heritability (*h* ^2^) for full and training datasets from pedigree-based and single-step analyses of mastitis and somatic cell score.

**Table 4:** **Cross-validation summary statistics for each single-trait model for mastitis**

**Data without daughter restrictions**			
	**AICC**	$\sum \chi ^{2}$	**WP**
Pedigree-based	-2.20	963462	0.109
BayesA	-2.13	966992	0.110
Single-step	-2.18	963280	0.111
**Data with daughter restrictions**			
	**AICC**	$\sum \chi ^{2}$	**WP**
Pedigree-based	-5.05	1846303	0.017
BayesA (non-weighted)	-4.82	1934351	0.009
BayesA (weighted)	-4.73	1839123	0.019
Single-step	-5.03	1787162	0.033

Corrected AIC (AICC) estimated via local weighted regression of average mastitis incidence per sire on EBV of sire for each model fit with the full dataset. Sum of *χ* ^2^ ($\sum \chi ^{\text {2}}$) is a measure of predictive ability, with smaller values being preferred. Median proportion of wrong predictions represented by WP.

Bivariate analyses allowed estimation of correlations between traits. Genetic correlations between liability to mastitis and SCS were 0.63 (*SD*=0.17) in the pedigree-based analyses using full *DATA*_*dtr*_ and 0.77 (*SD*=0.19) using training *DATA*_*dtr*_. Genetic correlations between liability to mastitis and SCS were very similar in the single-step analysis at 0.67 (*SD*=0.16) for the full *DATA*_*dtr*_ and 0.71 (*SD*=0.16) in the training *DATA*_*dtr*_. Correlation estimates were similar to a previously reported estimate of 0.62 (*SD*=0.03) [50]. Bivariate analyses were also performed using the HD genotype data. All estimates of variance components were similar to those obtained with the 50K genotype data. Because similar results were obtained, further analyses used 50K genotype data only.

**Figure 1:**
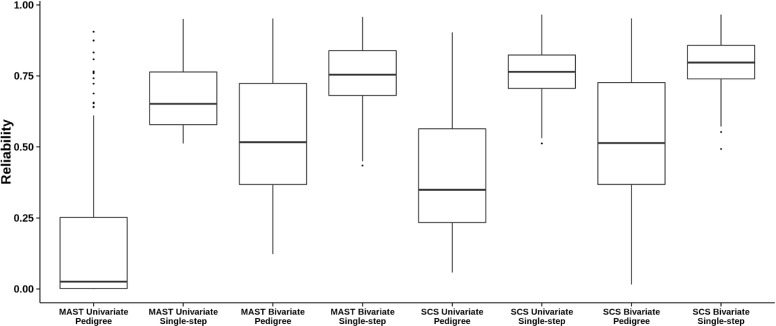
Reliability of sire EBV. Reliabilities obtained from pedigree-based and single-step univariate and bivariate analyses of mastitis (MAST) and somatic cell score (SCS).

**Figure 2:**
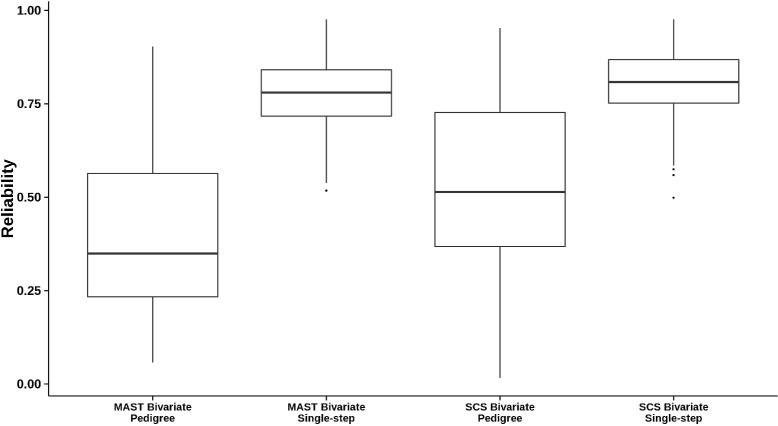
Reliability of sire EBV obtained with HD genotypes. Reliabilities obtained from pedigree-based and single-step bivariate analyses of mastitis (MAST) and somatic cell score (SCS) using HD genotypes.

Changes in reliability from pedigree-based models to single-step models were investigated for all sires included in *DATA*_*dtr*_. Reliabilities for univariate pedigree-based analysis of mastitis ranged from 0.01 up to 0.90. Average reliability for these sires was equal to 0.16. Reliabilities for bivariate pedigree-based analysis of mastitis and SCS ranged from 0.16 to 0.90, with average reliability equal to 0.54 for mastitis. This increase in reliability was expected from the incorporation of SCS as a correlated trait with higher heritability. The largest increase in reliability occurred with incorporation of genomic data. Approximated mean reliabilities of mastitis were equal to 0.68 and 0.80 in univariate and bivariate single-step analyses, respectively. A similar increase was observed for SCS, as shown in Figure 1. Changes in reliability were also explored using HD genotype data and are in Figure 2. Average reliability of mastitis was equal to 0.81 in bivariate single-step analyses (Figure 2).

Although not comparable to the other analyses, a measure of reliability was also estimated for BayesA analyses. Reliabilities of mastitis in the univariate BayesA analysis were equal to 0.22 in *DATA*_*dtr*_ and 0.23 in *DATA*_*full*_. In the bivariate BayesA analysis, reliability increased to 0.37. It must be acknowledged, however, that the above reliability values were calculated without incorporation of additional data sources, such as parental average or progeny data. It is expected that reliability will improve upon “blending” DGV to obtain genomic EBV (GEBV). Bivariate BayesA analysis also allowed us to calculate the correlation between marker effects for MAST and SCS. Genetic correlation between the two traits was equal to 0.22, which is lower than expected, however, may result from the highly polygenic nature of complex traits. It has been noted that multiple-trait models were better able to capture genetic correlation between traits when major QTL were present, as compared to traits with a more polygenic architecture [28].

### Model comparison

#### Predictive ability

Predictive ability of each model was assessed by the sum of *χ*^2^ values and the proportion of wrong predictions for mastitis incidence, where smaller values indicate better predictive ability. Values for each model are in Table 4, with *DATA*_*full*_ in the top portion and *DATA*_*dtr*_ in the bottom portion of the table. Prediction of mastitis incidence was estimated for 35 sires having at least 30 daughter records in the training data and at least 15 daughter records in the validation data. Each model’s *χ*^2^ value is in Table 4. The sum of *χ*^2^ values was smallest with the single-step analysis, which thus has the best predictive ability, followed by pedigree analysis and BayesA analysis, respectively. This was also observed for *DATA*_*full*_. BayesA analysis without weighting sire EBV had the poorest predictive ability. Thus, it was not included in further analyses. All models had very small values for median proportion of wrong predictions, ranging from 0.009 to 0.033.

#### Model fit

Goodness of fit for each model was evaluated by fitting a local weighted regression (LOESS) model between EBV obtained from the full dataset and mean incidence calculated for each sire in the full dataset. The best smoothing parameter was selected using a corrected AIC criteria (AICC) [44]. Smaller values of AICC are preferred, which were found for single-step and pedigree-based models, as shown in Table 4. Table 5 includes cross-validation summary statistics for each bivariate model. Again, the single-step model had the best model fit with genomic data, since it had the smallest AICC value. However, it also had the largest *χ*^2^ value. Correspondingly, the bivariate BayesA model had the smallest proportion of wrong predictions. In general, all single-trait models had comparable fits.

**Table 5:** **Cross-validation summary statistics for each bivariate model for mastitis and somatic cell score**

	**AICC**	$\sum \chi ^{2}$	**WP**
Pedigree-based	-4.77	1795125	0.02
Single-step	-4.76	1803782	0.02
Bivariate BayesA	-4.55	1947319	0.008

Corrected AIC (AICC) estimated via local weighted regression of average mastitis incidence per sire on EBV of sire for each model fit with the full dataset. Sum of *χ* ^2^ ($\sum \chi ^{\text {2}}$) is a measure of predictive ability, with smaller values being preferred. Median proportion of wrong predictions represented by WP.

When selecting a genomic evaluation method, there are many aspects to consider. Lowly heritable traits will need a larger number of records to reach reliabilities equivalent to those found for more highly heritable traits [51]. We acknowledge that the strict editing parameters used here do not reflect the true structure of the data. This was performed, however, in an effort to obtain a very clean dataset that would allow prediction with as little bias as possible. Completion of analyses with data without strict editing confirmed that results were comparable. This also provides an example of genomic prediction for traits with a limited number of available records. As more records are collected, it is also important that they remain consistent [52]. Consistent recording of health data is more difficult than other traits due to subjectivity of diagnosis and reporting. The size of the training populations used to estimate genetic effects in two stage methods will also increase as more data are collected. Accumulation of more health records over time, as well as additional genotypes, is expected to improve genomic prediction regardless of the method used. This will allow more rapid genetic improvement for lowly heritable, yet economically important traits.

Irrespective of the type of data, all genomic methodologies have benefits and disadvantages that must be considered prior to implementation. Bayesian approaches can incorporate prior knowledge about marker variances in the analysis [7], as well as determine which markers can be removed to decrease excess noise. Multi-step methods follow similar procedures to those already implemented for genetic evaluations and only minor modifications are needed to predict genomic values for young genotyped animals [13]. They also tend to be more computationally tractable as datasets grow larger [27], but require multiple steps to be performed prior to incorporation of genomic data. Deregression may need to be performed initially, which may produce spurious results, especially for lowly heritable traits and for individuals with low reliability estimates [12,14]. Resulting DGV from multi-stage analyses need to be blended with additional data if GEBV are desired.

One of the advantages of the single-step methodology, aside from only requiring one step, is that traditional BLUP methodology can be used by modifying only the relationship matrix. This makes the single-step method easy to implement for complex data and models such as multivariate, threshold, and random regression models [16]. A disadvantage of the single-step method is that it can be more computationally expensive due to having to form the **H**^−1^ matrix, although other methods have been developed to more efficiently compute this matrix [16]. Reliabilities of prediction also have to be approximated because direct matrix inversion is not feasible for large datasets. This will become especially important as the number of genotyped animals increases [23].

A straight-forward approach to extend multi-stage methods to multivariate models is lacking and requires further research. Performance of multivariate models will depend on the genetic architecture of traits and this must be considered [28]. Currently, the single-step method can be more readily applied to multiple traits, especially for traits with low heritability and reliability.

## Conclusions

Genomic data improves our ability to predict animal breeding values. The performance of specific genomic methods when implemented with real data will depend on many factors. Heritability of traits and reliability of genotyped individuals will have an impact on effectiveness of genomic evaluation methods. In this study, the differences between methodologies are probably due to many factors, including low heritability, use of a threshold sire model, and small training population size. Single-step models had the best predictive ability; BayesA models had the smallest proportion of wrong predictions. Given the current characteristics of producer-recorded health data, the single-step method provided several advantages compared to two-stage methods. As more health records are collected, the two methods are expected to perform more similarly.
